# Alexander Disease

**DOI:** 10.5334/jbsr.2588

**Published:** 2021-10-21

**Authors:** Laura Hartog, Sven Dekeyzer, Stephanie Vanden Bossche

**Affiliations:** 1 Universiteit Antwerpen, BE; 2 Universitair Ziekenhuis Antwerpen, BE

**Keywords:** Alexander disease, leukodystrophy, Canavan disease, megalencephalic leukoencephalopathy with subcortical cysts, macrocephaly

## Abstract

**Main teaching point:** The main differential diagnosis of leukodystrophy associated with macrocephaly consists of Alexander disease, Canavan disease, and megalencephalic leukodystrophy with subcortical cysts. Distinguishing imaging characteristics of Alexander disease are an apicoposterior gradient of white matter involvement and a periventricular T2-hypointense rim.

## Case

A 13-month-old girl was admitted to the pediatric ward because of failure to thrive, feeding difficulties and psychomotor regression. Clinical examination showed motor development delay, asymmetrical patellar tendon reflexes, positive Babinski sign, increased muscle tone in the upper limbs and macrocephaly. Urine analysis revealed increased levels of N-acetyl aspartate (NAA).

**Figure 1:**
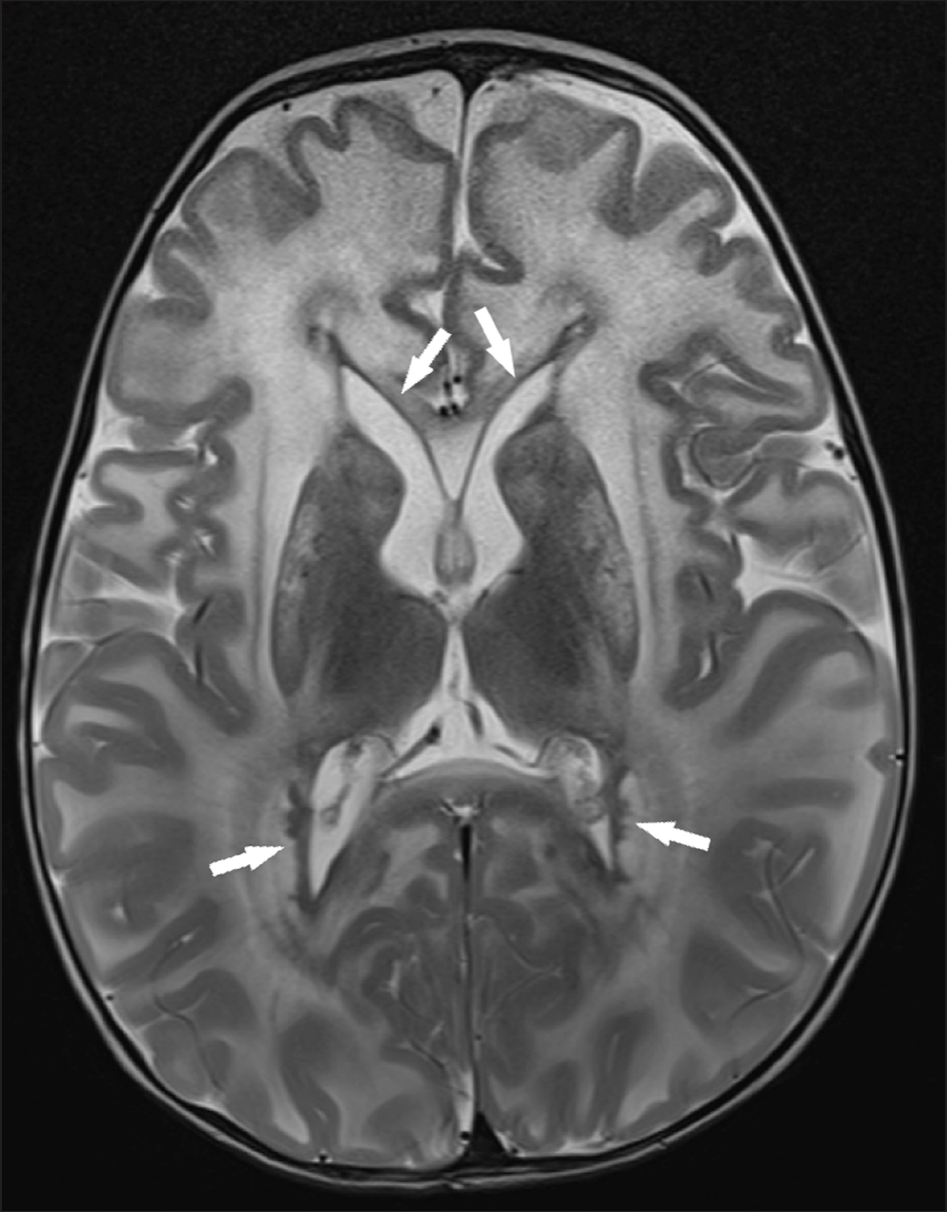


**Figure 2:**
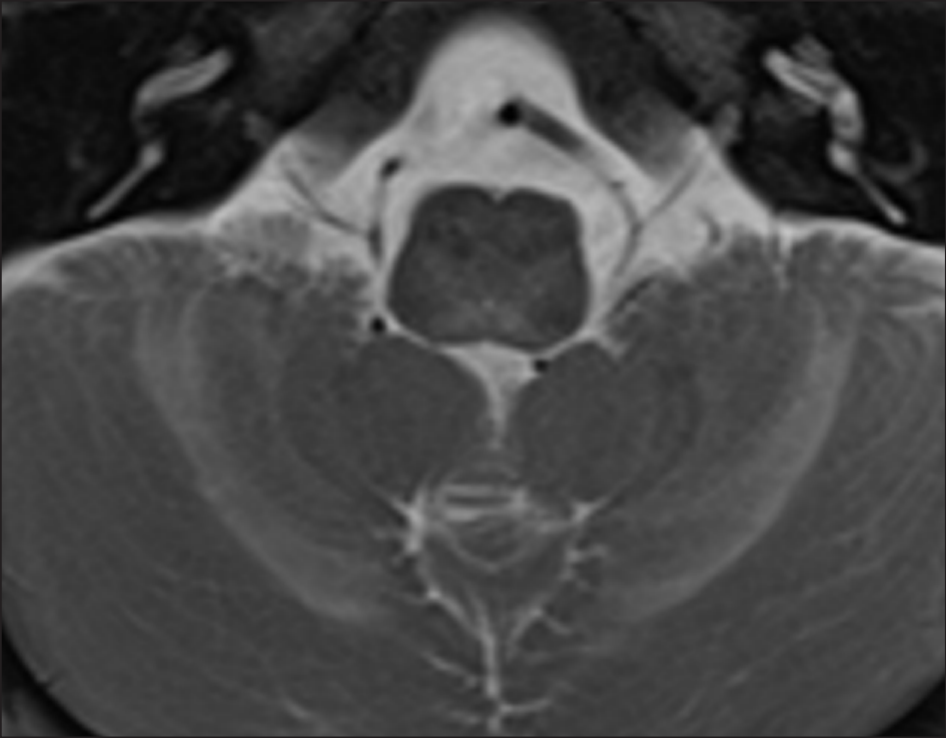


A Magnetic Resonance Imaging (MRI) of the brain was performed. T2-weighted images showed diffusely increased signal intensity of the supratentorial white matter, predominantly in the frontal lobes (***Figure 1***). Heterogeneous T2-signal changes are also present in the thalami and basal ganglia (***Figure 1***), and in the brainstem and cerebellum (***Figure 2***). Around the lateral ventricles a T2-hypointense periventricular rim is visible (arrows in ***Figure 1***).

Based on the presence of an apicoposerior gradient of supratentorial white matter involvement as well as a periventricular rim, a diagnosis of Alexander disease was suggested, which was confirmed genetically by the identification of a mutation in the GFAP-gene.

## Comment

Leukodystrophies are a heterogeneous group of genetic white matter disease. Their clinical and radiological characteristics are often non-specific and diagnosis can be difficult. The presence of macrocephaly is a helpful clinical clue, as the differential diagnosis of leukodystrophy with macrocephaly is limited to Canavan disease, megalencephalic leukoencephalopathy with subcortical cysts (MLC) and Alexander disease. Although these leukodystrophies all manifest with diffuse white matter changes, certain radiological characteristics can help coming to a correct diagnosis.

Canavan disease is associated with a pathological accumulation of NAA in the brain, resulting in an increased NAA peak on MR spectroscopy [1]. This patient’s urine NAA-level was elevated, but not as high as would be expected in Canavan disease, however. On MR spectroscopy (images not shown) an increased NAA-peak was absent. Furthermore, basal ganglia abnormalities in Canavan disease typically involve the globi pallidi and thalami, whereas in our patient the caudate nuclei and putamina were involved. Lastly, white matter changes in Canavan disease are diffuse without an apicoposterior gradient.

MLC is typically associated with subcortical cysts in the anterior temporal and frontoparietal lobes [1]. Subcortical cysts were lacking in our patient, however. Furthermore, contrary to the findings in our patient, basal ganglia abnormalities and an apicoposterior gradient are lacking in MLC.

Alexander disease typically shows high T2-weighted signal changes of the white matter with a frontal preponderance and abnormalities in the caudate nuclei, globi pallidi, thalami and the brainstem. Contrast enhancement may be seen in these structures [1]. A periventricular hypointense rim on T2-weighted images may be seen as a result of Rosenthal fibers accumulation.
